# Case report: A case of perinodal atrial tachycardia and review of the relevant clinical anatomy surrounding the retroaortic node

**DOI:** 10.3389/fcvm.2023.1143409

**Published:** 2023-05-19

**Authors:** Anvi Raina, Nicholas Y. Tan, Olubadewa A. Fatunde, Samuel J. Asirvatham, Christopher V. DeSimone

**Affiliations:** ^1^ Department of Cardiovascular Medicine, Mayo Clinic College of Medicine, Rochester, MN, United States; ^2^ Department of Cardiovascular Medicine, Mayo Clinic College of Medicine, Phoenix, AZ, United States

**Keywords:** perinodal atrial tachycardia, supraventricular tachycardia (SVT), retroaortic node, catheter ablation, electrophysiology study

## Abstract

A 70-year-old female presented with incessant supraventricular tachycardia that was refractory to metoprolol and sotalol. ECG revealed a narrow complex tachycardia with a rate of 163 beats per minute with a short RP relationship. She had salvos of atrial tachycardia which led to a severe reduction in ejection fraction as noted on echocardiography and hemodynamic instability. An electrophysiological study was performed, and findings suggested this to be an atrial tachycardia with earliest activation in the perinodal area. Radiofrequency ablation was carried out along the septum and associated structures to surround this region including the right atrium, non-coronary sinus of Valsalva, and the left atrium (anterior wall outside of the right superior pulmonary vein) to isolate this area and surround the focus with ablation lesions. The patient has done well post-procedure and continues to do well without any recurrence on low-dose flecainide at 8 months.

## Introduction

Perinodal atrial tachycardias (ATs) make up a minority of supraventricular tachycardia (SVT) cases and outcomes data on patients with this arrhythmia is limited to a few case reports and case series. Furthermore, ablation in this area can be challenging due to variations in atrial septal anatomy, difficulty with maintaining catheter contact, and proximity to the conduction system. It is critical for the electrophysiologist to appreciate the anatomy of the atrial septum and the potential arrhythmogenic role of the retroaortic node (RAN) in tachycardias originating from and around this region.

## Case presentation

**Figure 1:**
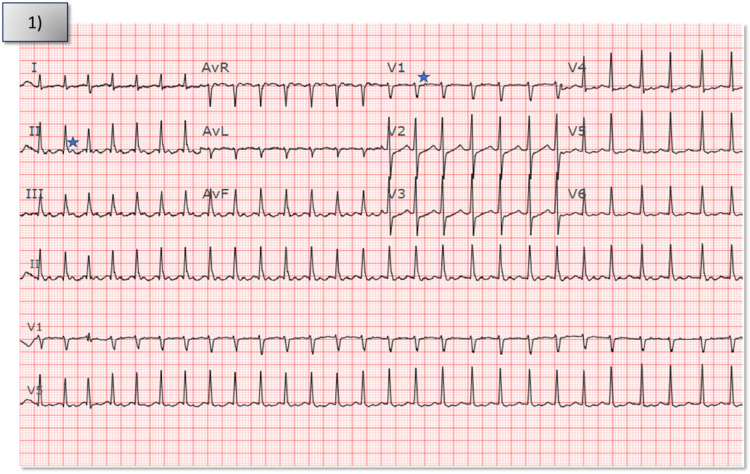
Index electrocardiogram from patient's presentation to the hospital showed narrow complex tachycardia with a short RP interval at a rate of 163 bpm. Star denotes *p* waves in V1 and lead II.

A 70-year-old female presented to the emergency department with recalcitrant palpitations and SVT. She noted a subacute onset of tachy-palpitations starting 2 months prior with accelerated frequency over the preceding week. She failed therapy with metoprolol and was admitted for sotalol loading given her symptoms. Her initial electrocardiogram showed a regular and narrow complex tachycardia with a rate of 163 bpm with a short RP relationship (Figure 1). The *p* wave morphology is biphasic in lead V1 (−/+), isoelectric in lead I, inferior directed axis, and narrower width when compared to sinus rhythm suggesting an origin in the atrial septum/perinodal region. The patient continued to have paroxysms of SVT during the hospitalization (other electrocardiograms and telemetry tracings shown in Supplementary Figures S1A,B). Echocardiography showed a preserved ejection fraction of 54% but a drastic decline in ejection fraction during with salvos of SVT. Given her symptoms, drop in ejection fraction during SVT, and failed drug therapy, she was offered an electrophysiologic study and ablation attempt.

### Electrophysiologic study

Diagnostic catheters were placed in the high right atrium, coronary sinus, the bundle of His, and the right ventricle. At baseline, she had salvos of narrow complex tachycardia which were poorly tolerated hemodynamically. Initially, the earliest atrial signal appeared to be on the His catheter with an atrial cycle length of 300 milliseconds (ms) with 1:1 conduction to the ventricle. Changes in the atrial-atrial interval preceded and dictated changes in the ventricular-ventricular interval; this wobble in the atrial cycle length was suggestive of AT. Continued hemodynamic instability during tachycardia made further diagnostic maneuvers difficult to execute. Fine mapping was performed with a multi-polar catheter (OctaRay, Biosense Webster, Irvine, California) to map the earliest atrial electrograms. The earliest atrial signals were −27 ms ahead of the surface p wave and appeared to be along the septum, approximately 11:30 along the tricuspid annulus and around the superior limbus which appeared thick on imaging (Figure 3A).

**Figure 2:**
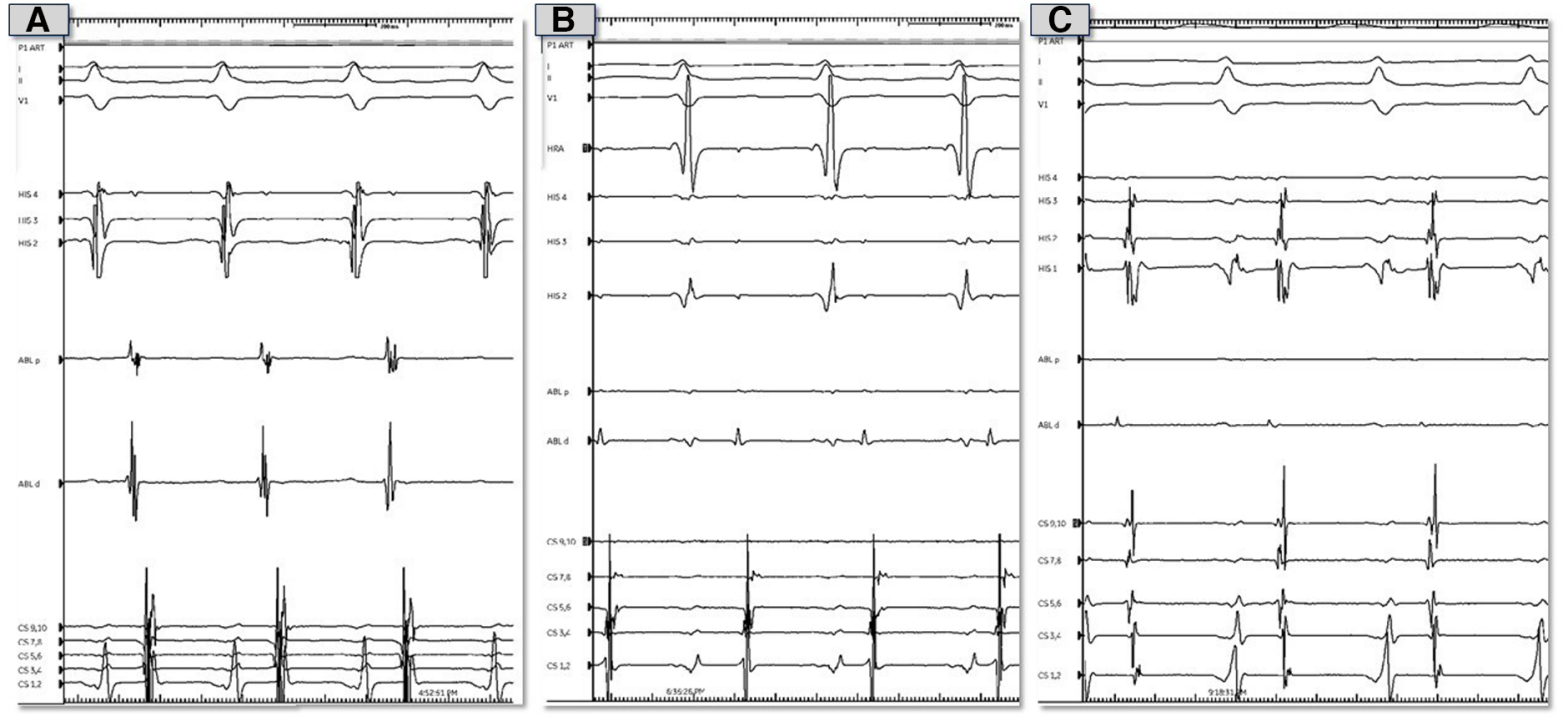
Representative electrocardiograms and intracardiac electrograms from electrophysiology study prior to ablation. (**A**) Tracings during tachycardia from theright atrial septum. (**B**) Tracings during tachycardia from the non-coronary sinus of Valsalva. (**C**) Tracings during tachycardia from the left atrial septum.

**Figure 3:**
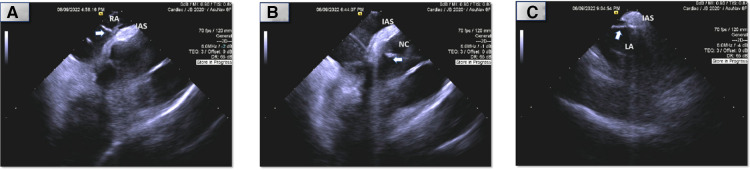
Intracardiac (ICE) echocardiography of ablation sites (arrow: ablation catheter, RA, right atrium; NC, non-coronary sinus of Valsalva; LA; atrial septum; IAS, interatrial septum). *Images are placeholders for videos which are attached. (**A**) Right atrial septum. (**B**) Non-coronary sinus of Valsalva. (**C**) Left atrial septum.

Using an irrigated catheter (ThermoCool SmartTouch, Biosense Webster, Irvine, California), ablation was performed at the earliest site (Figure 2A shows electrograms during tachycarida, Supplementary Figure S2A shows same site during sinus rhythm) using radiofrequency energy of 30–40 Watts with 30 cc/minute flow for 30–90 s per lesion. We noted almost immediate suppression of the tachycardia with ablation (Figure 3A shows catheter position during ablation with intracardiac echocardiography; Supplementary Figure S3A shows corresponding fluoroscopic position). We then performed additional lesions around this region, in particular targeting the muscle bundle opposing the non-coronary aortic sinus of Valsalva and superior limbus. Despite extensive ablation around this area, the tachycardia continued with a similar cycle length.

We then performed mapping of the opposite side of the septum in the non-coronary sinus of Valsalva which was not particularly early. We did position our catheter slightly higher in order to be adjacent to the prior lesions from the right atrium and noted atrial electrograms −17 ms ahead of the surface *p* wave (Figure 2B shows electrograms during tachycarida, Supplementary Figure S2B shows same site during sinus rhythm). Radiofrequency lesions were performed in this region at 30–40 Watts and 30 cc/min flow for 30–60 s per lesion (Figure 3B and Supplementary Figure S3B shows corresponding intracardiac echocardiography and fluoroscopic position of ablation catheter). Despite this, the tachycardia persisted, and we returned to the right atrium and performed additional radiofrequency ablation lesions in the same location along the septum as before. We noted transient suppression of tachycardia but afterwards we noted recurrence. Despite the use of a long sheath, some catheter movement was noted during ablation. To achieve better stability, we exchanged the radiofrequency ablation catheter for a cryoablation catheter (Medtronic CryoCath LP, Pointe-Claire, Canada), and created several lesions (freeze-thaw-freeze cycles to −80° Celsius for 3–4 min per lesion) along the superior limbus region. Despite initial suppression, AT recurred thereafter. This prompted transseptal access to map the left atrium. Mapping of the atrial septum was performed which revealed earlier (−29 ms relative to surface *p* wave) and fractionated signals near the left side of the superior limbus (Figure 2C shows electrograms during tachycarida, Supplementary Figure S2C shows same site during sinus rhythm) and close to the right superior pulmonary vein along the roof (directly adjacent to the area where right atrial ablation was performed). A series of ablation lesions were created using radiofrequency energy at 30–35 Watts and 30 cc/min flow for 30–60 s per lesion (Figure 3C shows corresponding ablation catheter position with intracardiac echocardiography; Supplementary Figure S3C shows corresponding fluoroscopic position). We noted temporary suppression and slowing of the tachycardia.

Given the extensive ablations already performed, we elected to end the procedure given the likelihood of automaticity from extensive ablation in the perinodal area. Immediately post-procedure she had salvos of SVT, prompting initiation of flecainide, metoprolol, and amiodarone bolus/drip overnight to allow the lesions and peri-ablation automaticity to settle. The patient recovered well from the procedure without any complications and was dismissed on flecainide and metoprolol. At her 3-month follow-up, she reported no further arrhythmia episodes and was tolerating flecainide without any ill effects and had reduced both flecainide and metoprolol dosing. At 8 months, she continues to do well without any recurrence and was offered to stop medications, but she preferred to stay on low doses given tolerance and improved symptoms as an added safeguard.

## Discussion

We present a unique case of a 70-year-old female with recalcitrant SVT. The patient suffered from salvos of SVT which although short lived were poorly tolerated hemodynamically. Electrophysiologic study suggested perinodal AT to be the mechanism and ablation was carried out from the right atrium, non-coronary sinus of Valsalva, and the left atrium to consolidate lesions in the area of interest.

### Relevant anatomy and arrhythmogenic role of the retroaortic node

The “true” atrial septum is limited to the floor of the fossa ovalis and its immediate muscular rims which are confluent with the apical portion of the triangle of Koch (1). This “true” or primum septum arises from the roof of the left side of the atrium and overhangs but does not reach the endocardial cushions. The roof of the right atrium then invaginates, resulting in an inner layer of adipose tissue between the myocardial layers, forming the septum secundum (2). After birth, left atrial pressure increases and pushes the septum primum against the septum secundum forming what we know clinically as the interatrial septum (3). The interatrial septum can serve as a potential arrhythmogenic focus for ATs and even facilitate tachycardias with intraseptal re-entry and atrial fibrillation (3). An increase in the thickness of the interatrial septum, often caused by fatty infiltration or fibrosis, is thought to be related to increased propensity for atrial arrhythmias (3, 4). Regardless, perinodal ATs remain a relatively less common focus accounting for 7%–13% of AT cases across different cases series (5–8).

**Figure 4:**
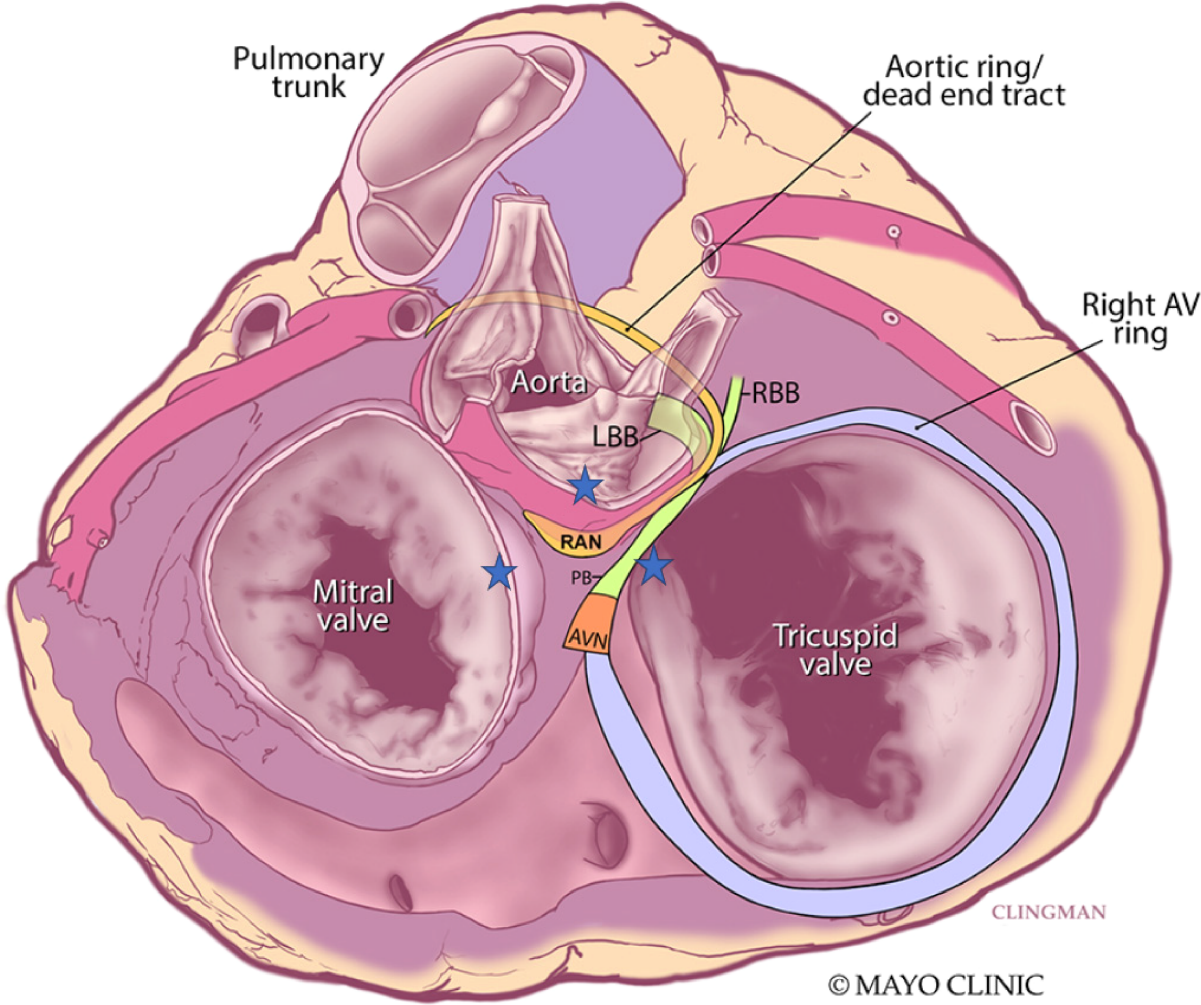
Superior view of the retroaortic node (RAN) and neighboring structures. Star represents vantage points of ablation to the area. AVN, atrioventricular node; PB, penetrating bundle; RBB, right bundle branch; LBB, left bundle branch. Adapted from Yanni et al. (14).

Despite some clinical and animal studies on arrhythmias arising from the interatrial septum, little attention has been placed on the arrhythmogenic role of the RAN, a large remnant of the “atrioventricular (AV) ring” in the atrial septum. The AV rings and RAN are thought to be remnants of the AV canal. The RAN is composed of cardiomyocytes and is separated from the AV node by the antero-superior rim of the fossa ovalis and from the bundle of His by the membranous septum (9, 10). Its location within the antero-superior interatrial wall means that it does have potential continuity with the transitional cells of the AV node but is distinct from the compact node itself (9) (Figure 4).

Electrophysiologic and molecular investigations of the RAN in animal studies suggest similar gene expression and ion channels as the compact AV node and point to its ability to serve as an ectopic source of ATs (11). Small clinical studies corroborate this notion with observations such as prompt termination of tachycardia with intravenous adenosine before production of AV block. In one such series of 43 patients, Bahora et al. (9) found all tachycardias (35/35) which were tested were found to be adenosine sensitive; the authors commented that this early termination was suggestive that the arrhythmic focus consists of node-like tissue but is distinct from the AV node itself. Clinical reports also suggest that the RAN may not respond to stress heating from radiofrequency ablation with automaticity (9, 12, 13). For ATs originating from this area, the right atrium offers a reasonable approach for ablation, although sometimes lesions need to be created from contiguous areas to “sandwich” the arrhythmic focus. The anterosuperior border of the fossa ovalis is marked by the non-coronary sinus of Valsalva and offers another vantage point for the proceduralist to approach perinodal ATs, including those tachycardias where the RAN may be implicated. While approaches from the right atrium and non-coronary sinus of Valsalva are well documented (12, 13), transeptal access and ablation of the left atrial septum must be considered in some patients where the septum is thicker, contact is not ideal, or atrial electrograms are not as early from the non-coronary sinus of Valsalva (Figure 4). In our case, ablation lesions were created from all three vantage points.

We must acknowledge some limitations in our case. Early termination of tachycardia in response to adenosine can be a clue for perinodal ATs. Unfortunately, due to the short-lived nature and hemodynamic instability of the tachycardia we were not able to test the effect of adenosine on the tachycardia in our patient. While cases of perinodal AT can be facile with ablation and termination from the right atrium or non-coronary sinus of Valsalva alone, we hope this report highlights a difficult case where different vantage points and energy sources had to be utilized. The use of unipolar recordings to corroborate the site of ablation could also be considered but was not done in our case. Lastly, the patient did have a short-lived episode of SVT post-procedure but has done well since suggesting durable lesion formation and the transient episode to be likely peri-ablation automaticity.

## Conclusion

Perinodal ATs are an infrequent cause of SVT, especially with a robust decline in ejection fraction, and have unique electrocardiographic characteristics. Understanding the anatomy of the atrial septum and the potential arrhythmogenic role of the retroaortic node is imperative for the electrophysiologist who tackles these rhythms. A stepwise approach and ablation from various vantage points should be considered in difficult perinodal AT cases.

### Patient perspective

Before treatment, the patient reported abrupt onset of symptoms with her tachycardia including palpitations, chest discomfort, and presyncope. She had to endure 6 ermegency room visits and two hospitalizations due to feeling extremely unwell due to SVT. Even when the episodes were short lived and self-terminated, she felt fatigued and out of energy for several hours afterwards. Particularly, during frequent salvos of tachycardia she felt unable to perform activities of daily living. After ablation, she is thankful for not having any more emergency room visits or even short runs of tachycardia at home. She was offered elimination of flecainide use, though she felt better keeping a low-dose as an added safeguard and tolerance.

## Data Availability

The original contributions presented in the study are included in the article/**Supplementary Material**, further inquiries can be directed to the corresponding author.
