# Supplementary figures and images for: Case report: A case of perinodal atrial tachycardia and review of the relevant clinical anatomy surrounding the retroaortic node
Source: Front Cardiovasc Med. 2023 May 19;10:1143409. doi: 10.3389/fcvm.2023.1143409 (PMC10235778; doi:10.3389/fcvm.2023.1143409)

## Slide 1
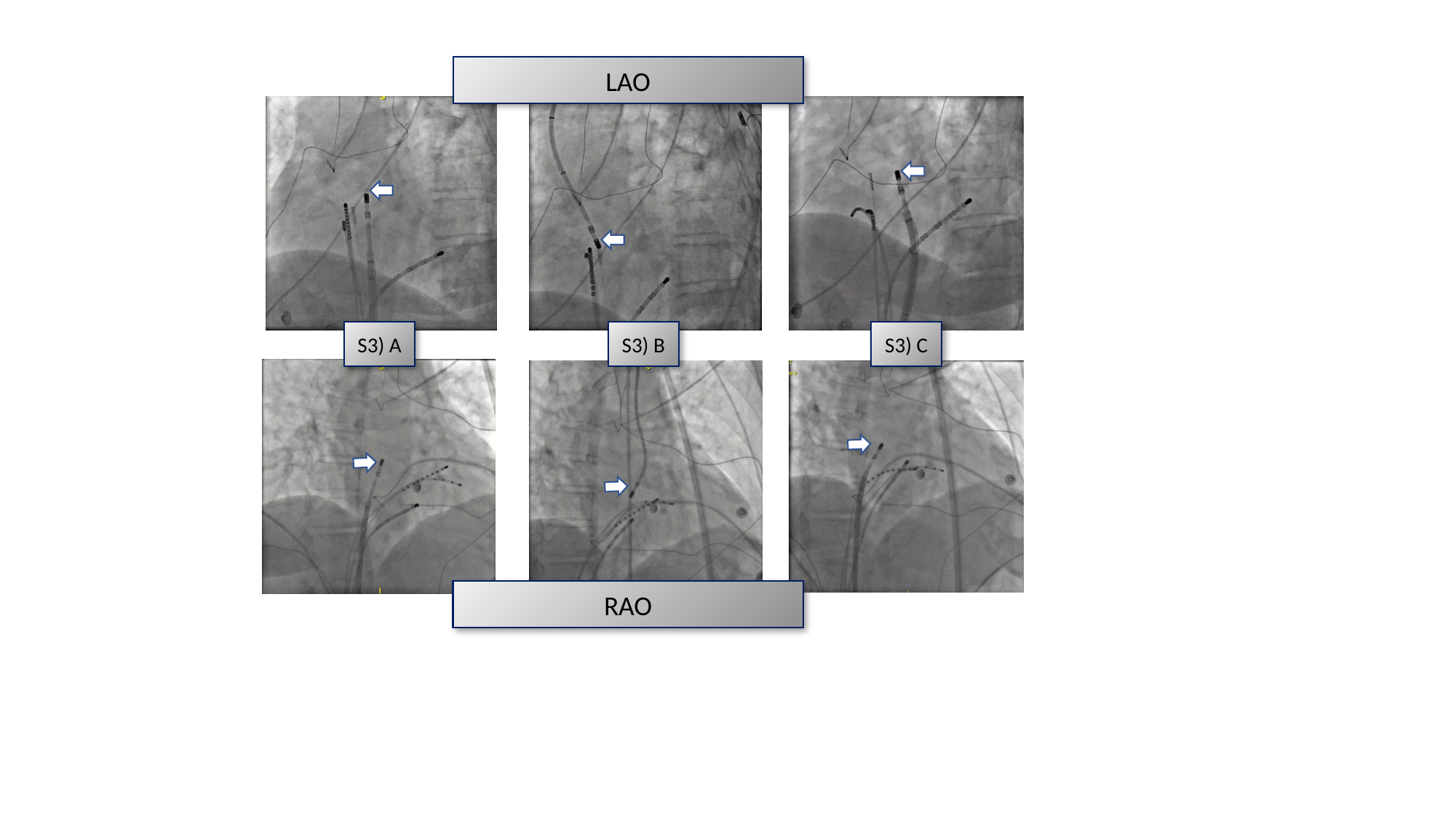

LAO
S3) B
S3) C
S3) A
RAO

Supplement: Supplementary file 1 [file Presentation1.pptx]

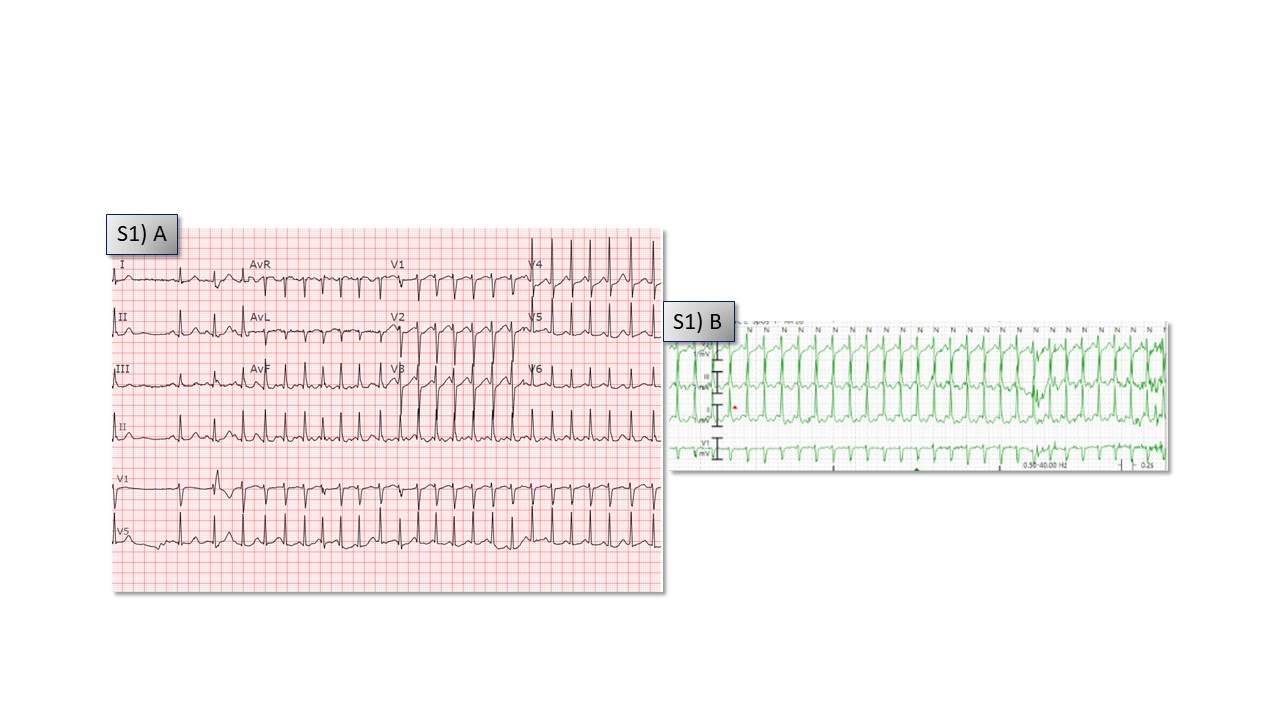

Supplement: Supplementary file 2 [file Image1.jpg]

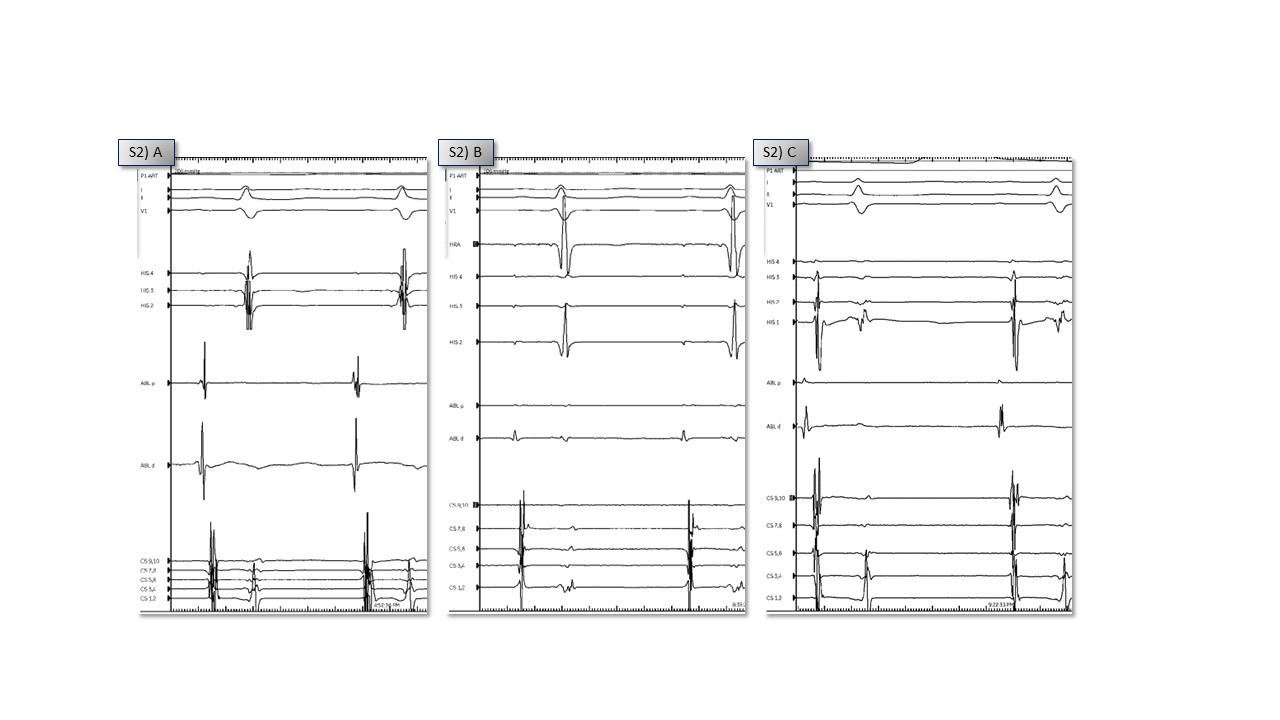

Supplement: Supplementary file 3 [file Image2.jpg]
